# Supplementary material for: Niche Partitioning with Temperature among Heterocystous Cyanobacteria (Scytonema spp., Nostoc spp., and Tolypothrix spp.) from Biological Soil Crusts
Source: Microorganisms. 2020 Mar 12;8(3):396. doi: 10.3390/microorganisms8030396 (PMC7142793; doi:10.3390/microorganisms8030396)
Supplement: Supplementary file 1 [file microorganisms-08-00396-s001.pdf]

Supplementary Materials

Niche Partitioning with Temperature Among Heterocystous Cyanobacteria (*Scytonema* spp., *Nostoc* spp., and *Tolypothrix* spp.) from Biological Soil Crusts

Supplementary Tables

Table S1. Accession numbers for the main generic groups for *Scytonema* spp. *Nostoc* spp. and *Tolypothrix* spp. according to our taxonomic assignment using our own cyanobacterial reference tree CYDRASIL.

Cyanobacterial group	Accession number	Source
Scytonema spp.	IMGID 2804885508	JGI
	KF359680	NCBI
	JN565276	NCBI
	MG641905	NCBI
Nostoc spp.	IMGID 2617914188	JGI
	IMGID 2651622745	JGI
	MG641900	NCBI
	IMGID 2776645028	JGI
	FJ815291	NCBI
Tolypothrix spp.	MG641915	NCBI
	AM230669	NCBI
	KM199732	NCBI

Table S2. Outcome of enrichment cultures for nitrogen-fixing photoautotrophs (nitrogen and organic carbon free medium, in the light) using variously sourced biocrusts as inoculum as a function of the incubation temperature. Given are the number of colonies containing each cyanobacterial taxa of interest, as identified morphologically by microscopy inspection. "S" stands for *Scytonema* spp., "N" for *Nostoc* spp., and "T" for *Tolypothrix* spp.

Inoculum origin	Replicate Enrichment	Incubation Temperature (°C)								
		4			25			30		
		S	N	T	S	N	T	S	N	T
Cold desert - sandy clay loam soil	1	0	7	9	14	15	10	32	25	0
	2	1	3	6	12	12	14	27	10	1
	3	0	4	12	11	18	9	33	27	3
Cold desert - clay loam soil	1	1	9	10	13	12	10	20	16	3
	2	2	6	7	15	9	12	17	0	0
	3	1	8	9	10	13	14	20	27	3
Hot desert - loamy sand soil	1	1	3	5	8	11	9	10	0	0
	2	0	3	6	11	10	10	40	0	0
	3	1	6	4	10	9	9	1	0	0
Hot desert - clay loam soil	1	0	2	7	15	6	12	40	0	0
	2	0	5	3	9	9	8	44	0	2
	3	1	5	5	10	12	10	39	1	0

Table S3. Cyanobacterial strains and their accession number in NCBI of their partial 16S rRNA sequence. Strain denominations include coding for the site of origin (HSN: cold desert sandy clay loam soil; HS: cold desert clay loam soil; FB: warm desert loamy sandy soil; JS: warm desert clay loam soil).

Cyanobacterial taxa	Strain	Accession number
Scytonema spp.	HSN006	MK487668
	HSN040	MK487667
	HS004	MK487662
	HS006	MK487664
	HS007	MK487669
	HS010	MK487673
	JS003	MK487663
	JS007	MK487665
	JS008	MK487672
	JS006	MK487670
	FB002	MK487671
	FB005	MK487666
Nostoc spp.	HSN008	MK487645
	HS020	MK487648
	HS002	MK487653
	HS094	MK487646
	HS096	MK487652
	HS013	MN815920
	FB21	MK487647
	FB23	MK487651
	FB25	MK487650
	FB26	MK487649
Tolypothrix spp.	HSN30	MK487655
	HSN031	MK487654
	HSN032	MK487658
	HSN034	MK487657
	HSN33	MK487656
	HSN042	MK487661
	JS100	MK487660
	FB100	MK487659

Table S4. Environmental biocrust surveys conducted at different locations around the world used in the meta-analysis, and the corresponding climate data. Raw sequences were downloaded from bacterial 16S rRNA tallies available publicly (see references). Environmental data was downloaded from WorldClim. "MAT" stands for mean annual temperature and "MTemWetQ" for mean temperature during the wettest quarter of the year (growth season).

Original location Descriptor	Latitude	Longitude	MAT	MTemp WetQ	Sequencing Platform	Reference (s)
Murcia, Carrascoy (Dark and light)	37.8	-1.3	16.6	13.3	Illumina	[1]
Albacete, Barrax Barrax (Dark and light)	39.0	-2.2	14.2	9.9		
Madrid, Campo Real (Dark and light)	40.3	-3.4	14.3	10.0		
Almeria, Amoladeras (Dark and light)	36.8	-2.2	17.8	15.3		
Almeria, Amoladeras (Light)	36.8	-2.2	17.8	15.3		
Navarra, Bardenas Reales (Light)	42.1	-1.4	14.3	16.0		
Alicante, Relleu (Dark and light)	38.5	-0.3	16.4	17.3		
Guadalajara, Zorita (Dark and light)	40.3	-2.8	14.0	9.7		
Cuenca, Huelves (Dark and light)	40.0	-2.9	13.2	8.9		
Huesca, Monegros (Dark and light)	41.9	-0.2	14.3	16.1		
Madrid, Morata (Light)	40.2	-3.4	14.5	10.2		
Madrid, Campo Real (Dark)	4.3	-3.4	13.8	9.5		
site17-Chihuahuan-WilcoxPlya	32.1	-109.9	16.5	25.0	454 Pyrosequencing	[2]
site8-NorthernGreatBasin-BlzdGap	42.1	-119.7	7.3	0.1		
site15-Sonoran-Chandler	33.3	-113.7	22.4	32.1		
site19-Mojave-CactusPln	34.1	-114.2	22.6	14.4		
site16-Sonoran-Dateland	32.8	-113.7	22.9	32.7		
site20-Mojave-SearlesLk	35.6	-117.4	19.7	9.5		
site13-Chihuahuan-FivePts	34.3	-106.8	13.2	23.0		
site22-Mojave-SodaLk	35	-111.8	8.6	17.5		
site11-NorthernGreatBasin-WhiteFlt	41.9	-118.9	9.5	0.7		
site18-Chihuahuan-Jornada	32.5	-106.7	15.2	24.1		
site14-Chihuahuan-SevilletaGyps	34.2	-106.8	13.4	22.9		
site10-NorthernGreatBasin-AlbertLk	42.1	-119.6	7.2	-0.1		
site21-Mojave-SodaLk	35.3	-116	21.2	12.4		
site5-ColoradoPlateau-Canyonlands	38.2	-109.7	12.2	19.2		

site3-ColoradoPlateau-GreenButte	38.7	-109.7	12.4	19.5		
site1-SonoranBatesW	32.2	-112.9	21.9	31.1		
site4-ColoradoPlateau-SundayChurt	38.6	109.6	7.7	19.9		
site2-ColoradoPlateau-SlickRock	38.6	-109.5	12.3	19.3		
site6-ColoradoPlateau-AcomaEx	35	-107.5	11.2	20.7		
site12-NorthernGreatBasin-CulverRd	44.5	-121.1	8.6	1.4		
site9-NorthernGreatBasin-AlvordHS	42.5	-118.5	9.3	8.2		
site7-ColoradoPlateau-ElMorro	35	-108.3	8.2	17.6		
Homburg, Goessenheim, Germany	50	9.8	9.1	16.0	Illumina	[3]
Tabernas, Almeria, Spain	37	-2.4	16.0	12.8		
Nat, Reserve Gynge Alvar, Sweden	56.5	16.4	7.5	15.3		
Hohe Tauern National Park, Austria	47	12.8	-1.8	5.5		
Cold Desert Silty - clay loam soil	41.1	-113.0	10.3	15.0	Illumina	[4,5]
Cold Desert - sandy clay loam soil	41.1	-113.0	10.1	14.8		
Hot Desert Silty - clay loam soil	32.5	-106.7	15.2	24.2		
Hot Desert Sandy - loamy sand soil	32.4	-105.9	16.2	25.0		
Desert, early-developed biocrusts (China)	44.8	88.2	7.1	24.0	454 Pyrosequencing	[6]
Moab, Green Butte site	38.7	-109.6	12.4	19.5	Illumina	[7]
Canastra National Park	-20.3	-46.6	19.8	21.7	Illumina	[8]
Capao National Park	-19.3	-43.5	19.1	20.8		
Cipo National Park	-19.3	-43.5	19.1	20.8		
Furnas National Park	-20.2	-47.4	20.9	22.5		
Vassununga National Park	-20.3	-46.3	20.3	22.4		
Zagaia National Park	-21.3	-47.6	21.6	23.5		
Blue gramma	34.3	-106.6	12.8	22.2	Illumina	[9]
Black gramma	34.3	-106.7	12.9	22.8		
MRME	34.3	-106.7	12.9	22.8		
Actopan	20.3	-98.92	16.8	18	Illumina	[10]
Atexcac	19.3	97.3	24.7	25.5		
Western Australia - ERR2940139	-29.2	116.7	20.0	13.8	Ion Torrent PGM	[11]
Western Australia - ERR2940142	-29.2	116.7	20.1	14.0		
Western Australia - ERR2940143	-29.2	116.7	20.1	14.0		

Western Australia - ERR2940148	-29.2	116.7	20.2	14.0		
Western Australia - ERR2940149	-29.2	116.7	20.2	14.0		
Western Australia - ERR2940151	-29.2	116.7	20.0	13.8		
Western Australia - ERR2940153	-29.2	116.7	20.0	13.8		
Western Australia - ERR2940163	-29.2	116.7	20.0	13.8		
Western Australia - ERR2940164	-29.2	116.7	20.2	14.0		
Western Australia - ERR2940165	-29.2	116.7	20.0	13.8		
Western Australia - ERR2940166	-29.2	116.7	20.0	13.8		
Western Australia - ERR2940168	-29.2	116.7	20.0	13.8		
Western Australia - ERR2940172	-29.2	116.7	20.2	14.0		
Western Australia -ERR2940173	-29.2	116.7	20.2	14.0		
Western Australia - ERR2940180	-29.2	116.7	20.0	13.8		
Western Australia - ERR2940187	-29.2	116.7	20.2	14.0		

Table S5. Full results for linear regression between relative proportions (arcsine transformed) of *Scytonema* spp. and mean annual temperature (MAT).

SUMMARY OUTPUT MAT

Regression Statistics	
Multiple R	0.385282827
R Square	0.148442857
Adjusted R Square	0.137663653
Standard Error	0.442982004
Observations	81

ANOVA

	df	*SS*	*MS*	*F*	*Significance F*
Regression	1	2.702369724	2.702369724	13.77122581	0.000382583
Residual	79	15.50241141	0.196233056		
Total	80	18.20478114			

	Coefficients	*Standard Error*	*t Stat*	*P-value*	*Lower 95%*	*Upper 95%*	*Lower 95.0%*	*Upper 95.0%*
Intercept	0.326979334	0.140353785	2.329679482	0.022377903	0.047612112	0.606346556	0.047612112	0.606346556
MAT	0.033574855	0.009047484	3.710960228	0.000382583	0.015566288	0.051583422	0.015566288	0.051583422

Table S6. Full results for linear regression between relative proportions (arcsine transformed) of *Scytonema* spp. and mean temperature during the wettest quarter of the year (MTempWetQ).

SUMMARY OUTPUT MTemWetQ

Regression Statistics	
Multiple R	0.567486955
R Square	0.322041444
Adjusted R Square	0.31345969
Standard Error	0.395258081
Observations	81

ANOVA

	df	*SS*	*MS*	*F*	*Significance F*
Regression	1	5.862694009	5.862694009	37.52629697	3.30881E-08
Residual	79	12.34208713	0.156228951		
Total	80	18.20478114			

	Coefficients	*Standard Error*	*t Stat*	*P-value*	*Lower 95%*	*Upper 95%*	*Lower 95.0%*	*Upper 95.0%*
Intercept	0.148905641	0.117230831	1.270191803	0.207744659	-0.084436492	0.382247773	-0.084436492	0.382247773
MTempWetQ	0.041408599	0.006759626	6.125871119	3.30881E-08	0.027953899	0.054863298	0.027953899	0.054863298

Table S7. Full results for linear regression between relative proportions (arcsine transformed) of *Nostoc* spp. and mean annual temperature (MAT).

SUMMARY OUTPUT MAT

Regression Statistics	
Multiple R	0.305397873
R Square	0.093267861
Adjusted R Square	0.081790239
Standard Error	0.285278919
Observations	81

ANOVA

	df	*SS*	*MS*	*F*	*Significance F*
Regression	1	0.661331882	0.661331882	8.126061379	0.005564008
Residual	79	6.429340888	0.081384062		
Total	80	7.09067277			

	Coefficients	*Standard Error*	*t Stat*	*P-value*	*Lower 95%*	*Upper 95%*	*Lower 95.0%*	*Upper 95.0%*
Intercept	0.605863038	0.090387365	6.70296159	2.74752E-09	0.425951488	0.785774589	0.425951488	0.785774589
MAT	-0.016609306	0.00582655	-2.850624735	0.005564008	-0.028206763	-0.005011849	-0.028206763	-0.005011849

Table S8. Full results for linear regression between relative proportions (arcsine transformed) of *Nostoc* spp. and mean temperature during the wettest quarter of the year (MTempWetQ).

SUMMARY OUTPUT MTemWetQ

Regression Statistics	
Multiple R	0.528227365
R Square	0.279024149
Adjusted R Square	0.269897872
Standard Error	0.254384435
Observations	81

ANOVA

	df	*SS*	*MS*	*F*	*Significance F*
Regression	1	1.978468936	1.978468936	30.57371165	4.01685E-07
Residual	79	5.112203834	0.064711441		
Total	80	7.09067277			

	Coefficients	*Standard Error*	*t Stat*	*P-value*	*Lower 95%*	*Upper 95%*	*Lower 95.0%*	*Upper 95.0%*
Intercept	0.751367363	0.075448675	9.958655448	1.3263E-15	0.601190531	0.901544195	0.601190531	0.901544195
MTempWetQ	-0.024055065	0.004350433	-5.52935002	4.01685E-07	-0.032714385	-0.015395745	-0.032714385	-0.015395745

Table S9. Full results for linear regression between relative proportions (arcsine transformed) of *Tolypothrix* spp. and mean annual temperature (MAT).

SUMMARY OUTPUT MAT

Regression Statistics	
Multiple R	0.234510246
R Square	0.054995055
Adjusted R Square	0.043032967
Standard Error	0.333940947
Observations	81

ANOVA

	df	*SS*	*MS*	*F*	*Significance F*
Regression	1	0.512691364	0.512691364	4.597446184	0.035094216
Residual	79	8.809807915	0.111516556		
Total	80	9.322499279			

	Coefficients	*Standard Error*	*t Stat*	*P-value*	*Lower 95%*	*Upper 95%*	*Lower 95.0%*	*Upper 95.0%*
Intercept	0.568785714	0.105805373	5.375773465	7.52141E-07	0.358185388	0.77938604	0.358185388	0.77938604
MAT	-0.014624121	0.006820425	-2.144165615	0.035094216	-0.028199837	-0.001048404	-0.028199837	-0.001048404

Table S10. Full results for linear regression between relative proportions (arcsine transformed) of *Tolypothrix* spp. and mean temperature during the wettest quarter of the year (MTempWetQ).

SUMMARY OUTPUT MTemWetQ

Regression Statistics	
Multiple R	0.326119109
R Square	0.106353673
Adjusted R Square	0.095041695
Standard Error	0.324739764
Observations	81

ANOVA

	df	*SS*	*MS*	*F*	*Significance F*
Regression	1	0.991482044	0.991482044	9.401862853	0.002966997
Residual	79	8.331017235	0.105455914		
Total	80	9.322499279			

	Coefficients	*Standard Error*	*t Stat*	*P-value*	*Lower 95%*	*Upper 95%*	*Lower 95.0%*	*Upper 95.0%*
Intercept	0.630149478	0.096315583	6.542549621	5.52005E-09	0.438438107	0.82186085	0.438438107	0.82186085
MTempWetQ	-0.017028812	0.005553636	-3.066245726	0.002966997	-0.028083047	-0.005974576	-0.028083047	-0.005974576

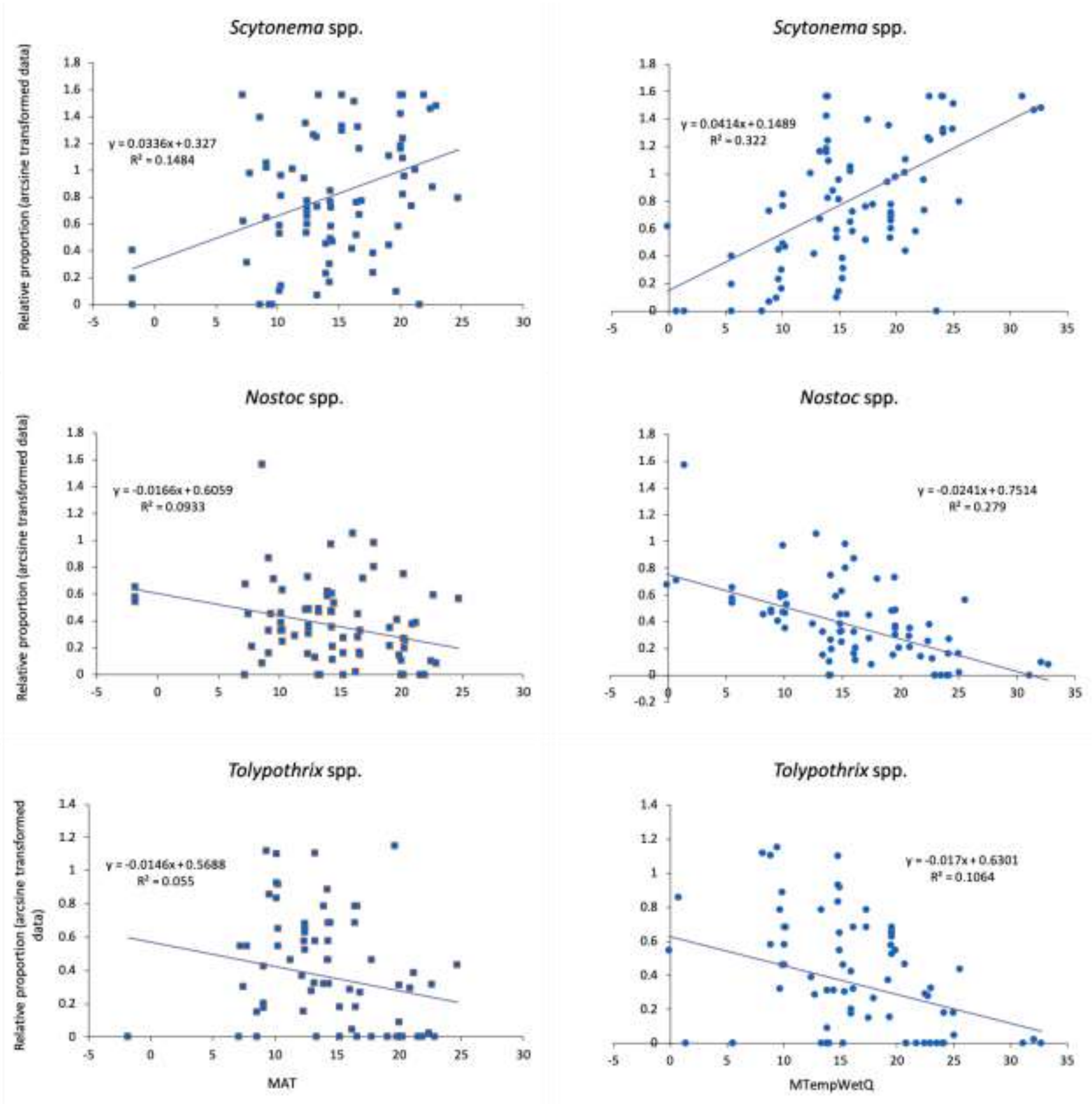

Figure S1. Linear regression between the proportion of sequence reads (arcsine transformed) of each taxon among heterocystous cyanobacteria and climatic parameters (MAT and MTempWetQ). MAT: Mean annual temperature, MTemWetQ: Mean temperature during the wettest quarter of the year.

References

1. Muñoz-Martín, M.Á.; Becerra-Absalón, I.; Perona, E.; Fernández-Valbuena, L.; Garcia-Pichel, F.; Mateo, P. Cyanobacterial biocrust diversity in Mediterranean ecosystems along a latitudinal and climatic gradient. *New Phytol.* **2018**, *221*, 123–141, DOI:10.1111/nph.15355.
2. Garcia-Pichel, F.; Loza, V.; Marusenko, Y.; Mateo, P.; Potrafka, R.M. Supp. material -Temperature drives the continental-scale distribution of key microbes in topsoil communities. *Science (80-.).* **2013**, *340*, 1574–1577, DOI:10.1126/science.1236404
3. Williams, L.; Loewen-Schneider, K.; Maier, S.; Büdel, B. Cyanobacterial diversity of western European biological soil crusts along a latitudinal gradient. *FEMS Microbiol. Ecol.* **2016**, *92*, 1–9, DOI:10.1093/femsec/fiw157.
4. Velasco Ayuso, S.; Giraldo Silva, A.; Nelson, C.; Barger, N.N.; Garcia-Pichel, F. Microbial Nursery Production of High-Quality Biological Soil Crust Biomass for Restoration of Degraded Dryland Soils. *Appl. Environ. Microbiol.* **2016**, *83*, 1–16, DOI: 10.1128/AEM.02179.
5. Bethany, J.; Giraldo-Silva, A.; Nelson, C.; Barger, N.N.; Garcia-Pichel, F. Optimizing the Production of Nursery-Based Biological Soil Crusts for Restoration of Arid Land Soils. *Appl. Environ. Microbiol.* **2019**, *85*, 1–13, DOI:10.1128/aem.00735-19.
6. Zhang, B.; Kong, W.; Nan, W.; Zhang, Y. Bacterial diversity and community along the succession of biological soil crusts in the Gurbantunggut Desert, Northern China. *J. Basic Microbiol.* **2016**, *56*, 670–679, DOI:10.1002/jobm.201500751.
7. Couradeau, E.; Karaoz, U.; Lim, H.C.; Nunes da Rocha, U.; Northen, T.; Brodie, E.; Garcia-Pichel, F. Bacteria increase arid-land soil surface temperature through the production of sunscreens. *Nat. Commun.* **2016**, *7*, 1–7, DOI:10.1038/ncomms10373.
8. Machado-de-Lima, N.M.; Fernandes, V.M.C.; Roush, D.; Velasco Ayuso, S.; Rigonato, J.; Garcia-Pichel, F.; Zanini Branco, L.H. The Compositionally Distinct Cyanobacterial Biocrusts From Brazilian Savanna and Their Environmental Drivers of Community Diversity. *Front. Microbiol.* **2019**, *10*, 1–10, DOI:10.3389/fmicb.2019.02798.
9. Fernandes, V.M.C.; Machado de Lima, N.M.; Roush, D.; Rudgers, J.; Collins, S.L.; Garcia-Pichel, F. Exposure to predicted precipitation patterns decreases population size and alters community structure of cyanobacteria in biological soil crusts from the Chihuahuan Desert. *Environ. Microbiol.* **2018**, *20*, 259–269, DOI:10.1111/1462-2920.13983.
10. Becerra-Absalón, I.; Muñoz-Martín, M.Á.; Montejano, G.; Mateo, P. Differences in the Cyanobacterial Community Composition of Biocrusts From the Drylands of Central Mexico. Are There Endemic Species? *Front. Microbiol.* **2019**, *10*, 1–21, DOI:10.1111/nph.15355.
11. Moreira-Grez, B.; Tam, K.; Cross, A.T.; Yong, J.W.H.; Kumaresan, D.; Nevill, P.; Farrell, M.; Whiteley, A.S. The Bacterial Microbiome Associated With Arid Biocrusts and the Biogeochemical Influence of Biocrusts Upon the Underlying Soil. *Front. Microbiol.* **2019**, *10*, 1–13, DOI:10.3389/fmicb.2019.02143.
